# Supplementary material for: Transcriptomic Profiling of DNA Damage Response in Patient-Derived Glioblastoma Cells before and after Radiation and Temozolomide Treatment
Source: Cells. 2022 Apr 4;11(7):1215. doi: 10.3390/cells11071215 (PMC8997841; doi:10.3390/cells11071215)
Supplement: Supplementary file 1 [file cells-11-01215-s001.zip › Supplementary Figures.pdf]

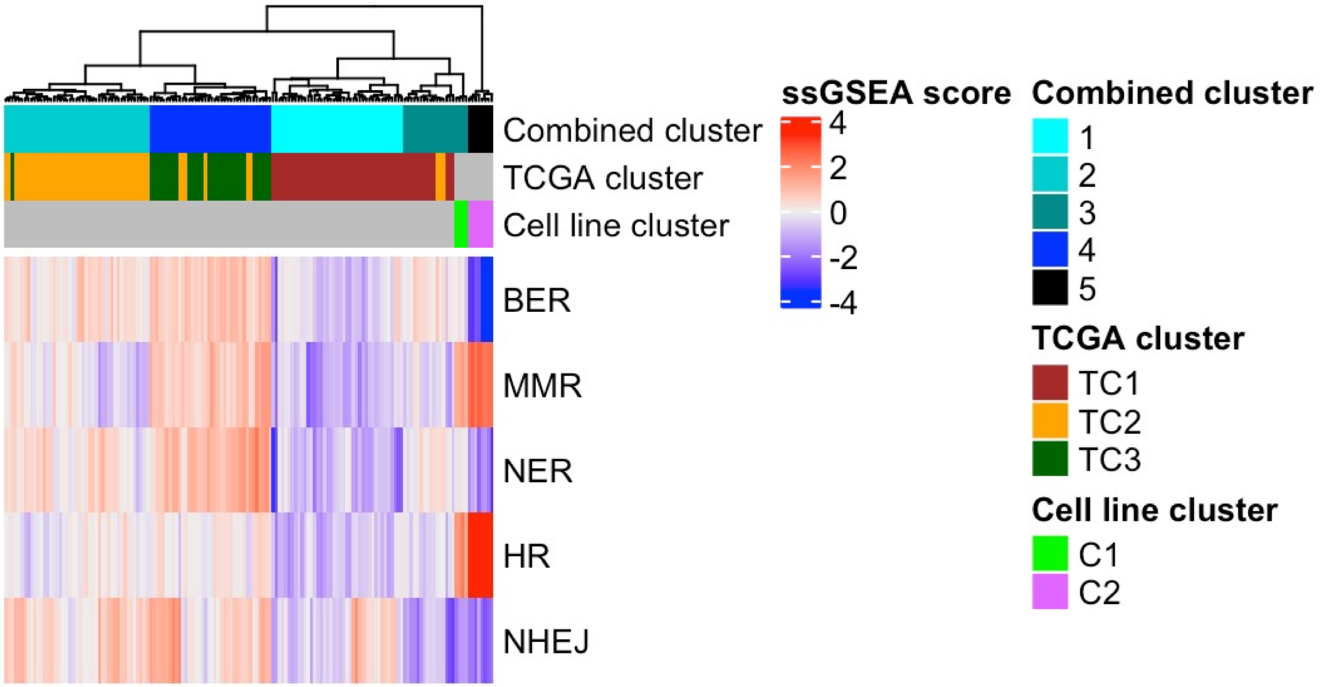

Figure S1. Combined hierarchical cluster of glioblastoma cell lines and TCGA patients. Hierarchical clustering was performed on ssGSEA log-transformed Z-scores of TCGA samples and cell lines, from which 5 clusters appeared. Combined clusters 2, 4 and 1 resembled similarity to the respective TCGA clusters TC2, TC3 and TC1. The Combined cluster 3 was a mix of predominantly TC1, as well as TC2 and four cell lines all belonging to C1. Combined cluster 5 was a distinct cluster containing the C2 cell lines.

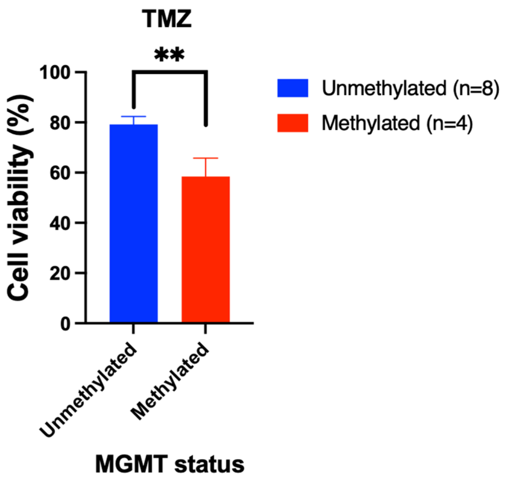

Figure S2. Cell viability of MGMT methylated and unmethylated glioblastoma cell lines treated with TMZ. Cell lines were grouped as either methylated (red) or unmethylated (blue) based on MGMT methylation status and the combined cell viabilities from respective cell lines, determined by MTT assay, were compared with a student's t-test. The comparison shows methylated MGMT glioblastoma cell lines having lower cell viability than unmethylated MGMT cell lines ($p < 0.01$). P-value < 0.05 was determined to be significant (** < 0.01).

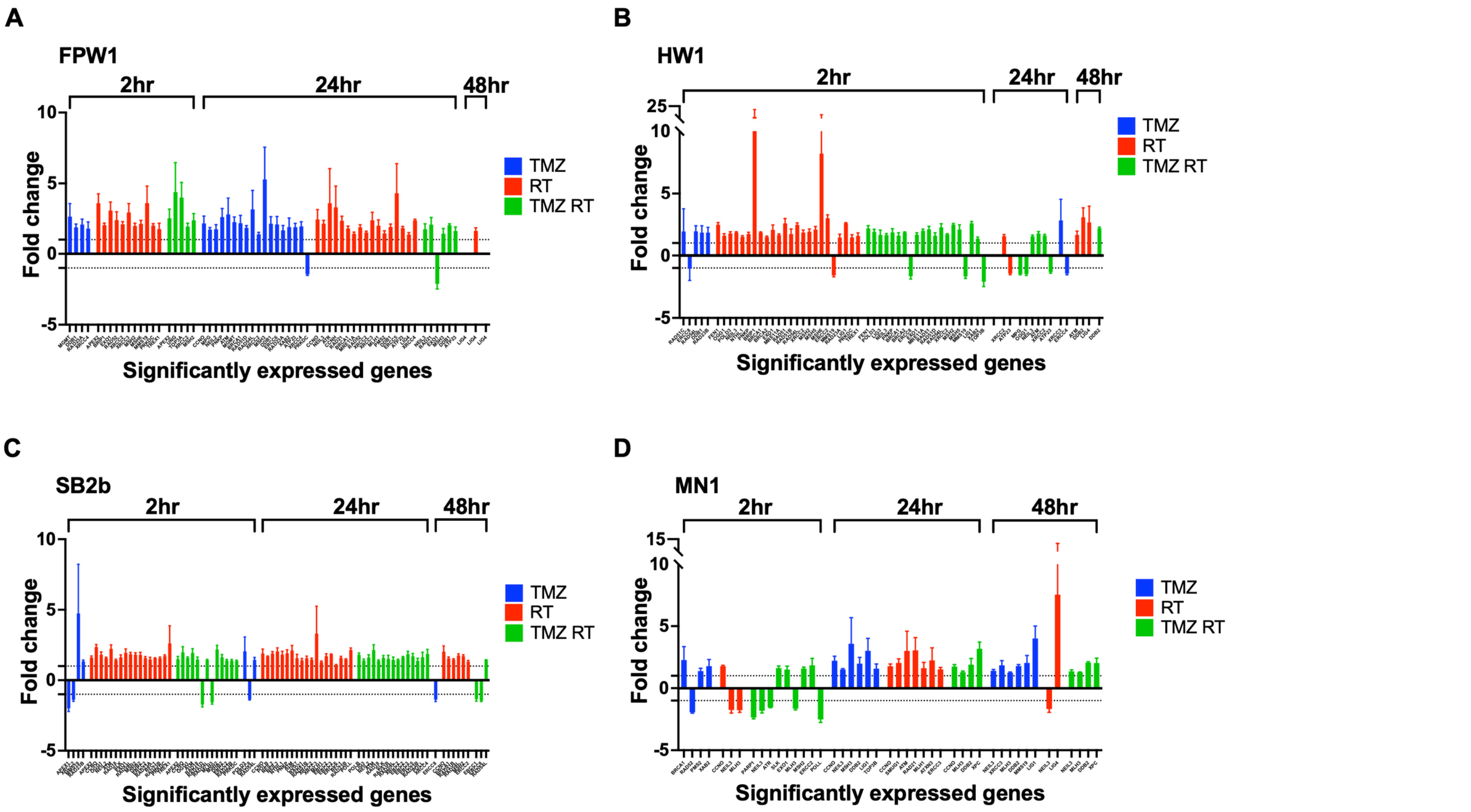

Figure S3. Distribution of DEGs across time points and treatments for FPW1 (**A**), HW1 (**B**), SB2b (**C**) and MN1 (**D**) glioblastoma cell lines. qPCR using a Custom TaqMan array card was undertaken to assess mRNA expression of 84 DNA repair genes, with DEGs identified as genes with significantly higher or lower expression than the untreated control. The fold-changes of DEGs are reported, across the 2hr, 24hr and 48hr time points for TMZ-, RT- and TMZ+RT-treated cells. Variability between the magnitude and timing of DDR gene changes occurred between cell lines, however a general trend appeared whereby an upregulation of DDR genes occurred predominantly within 24hr after treatment, especially in RT- and TMZ +RT- treated cells. This general trend is shared across FPW1, HW1 and SB2b cell lines whereas MN1 cells had a lower frequency of DDR gene changes .

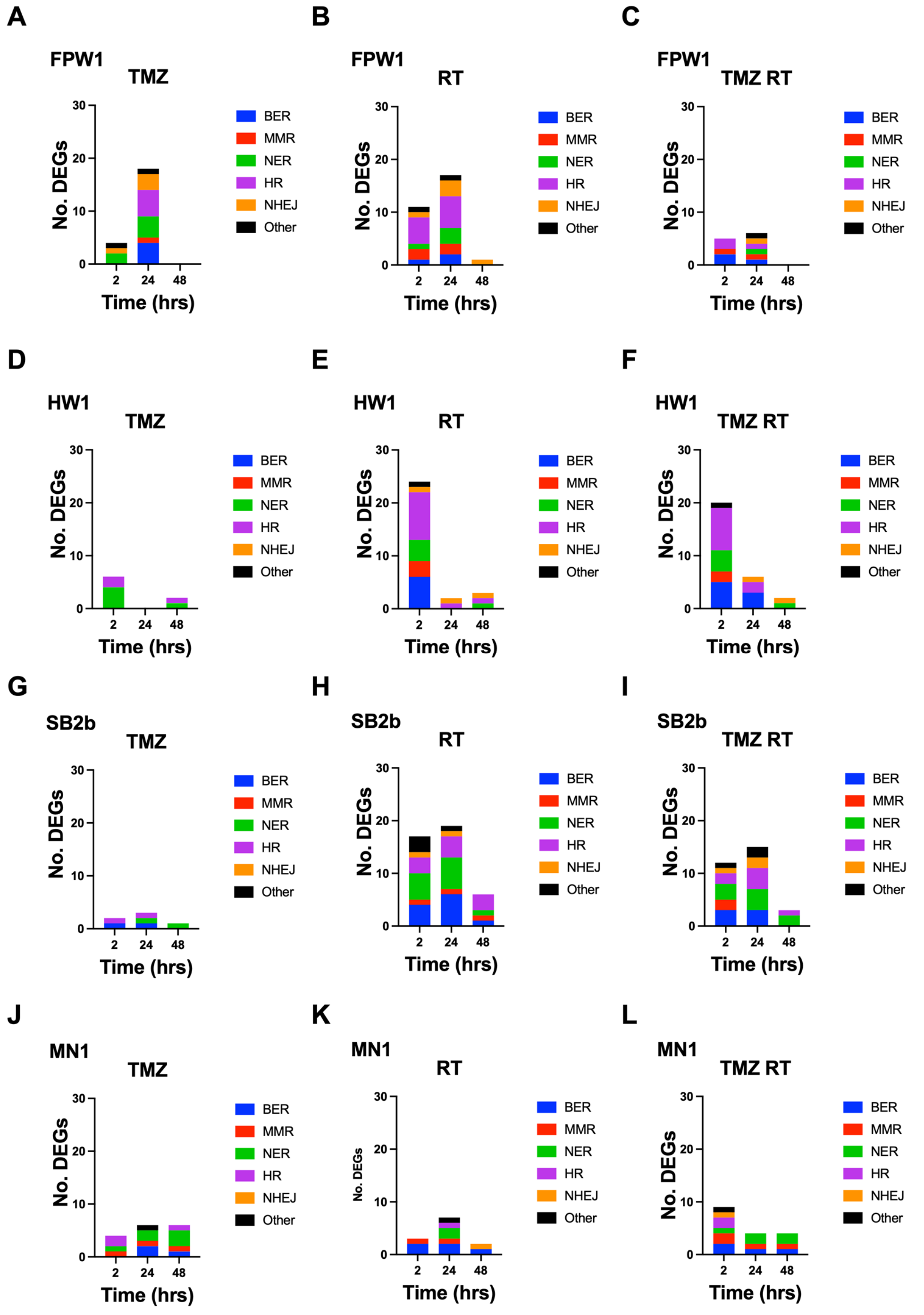

Figure S4. Frequency of DEGs per cell line (FPW1 (**A-C**), HW1 (**D-F**), SB2b (**G-I**), MN1 (**J-L**)), including the distribution of DDR pathways for each respective time point and treatment. Across the cell lines, TMZ induced changes in NER genes especially 2hr after treatment, while BER and other pathways became upregulated 24hr after treatment before a decrease in expression at 48hr in all cell lines, except for MN1. RT provoked an upregulation in all DDR pathways across the 2hr and 24hr timepoints, with a predominance in HR and NER genes, while the combination of TMZ+RT had similar DDR changes to RT-treated cells.
